# Emerging role of microRNA in hepatocellular carcinoma (Review)

**DOI:** 10.3892/ol.2014.2816

**Published:** 2014-12-19

**Authors:** JIN GONG, XING-XING HE, DE-AN TIAN

**Affiliations:** Institute of Liver Diseases, Tongji Hospital, Tongji Medical College, Huazhong University of Science and Technology, Wuhan, Hubei 430030, P.R. China

**Keywords:** microRNA, hepatocellular carcinoma, diagnosis, therapy

## Abstract

Hepatocellular carcinoma is a type of cancer characterized by significant morbidity and high mortality rates worldwide. Previous studies have revealed that alterations in microRNA (miRNA) expression are a common feature of cancer. Furthermore, as evolutionarily conserved, non-encoding RNAs, miRNAs have demonstrated fundamental roles in the various biological processes involved in cancer. Genome-wide miRNA expression profile studies and bioinformatic methods have provided comprehensive insight into the role of cancer-related miRNAs. In addition, investigation of the function and mechanisms of miRNAs has provided an understanding of the association with the pathogenesis of cancer. In the present review, the tumor-promoting or tumor-suppressive roles and underlying mechanisms of certain significant miRNAs at a single and integral level are summarized. Furthermore, the recognition of miRNA-gene networks and current advances in the potential use of miRNA-based diagnosis and therapy are discussed.

## 1. Introduction

Hepatocellular carcinoma (HCC) is one of the most common forms of malignancy worldwide, and is the second leading cause of cancer-related mortality (GLOBOCAN 2012) (1). In the majority of cases, HCC originates from chronic liver diseases, mainly with progression from hepatitis B virus (HBV) and hepatitis C virus (HCV) to cirrhosis in Asian populations. Additional common risk factors for HCC include alcohol abuse, exposure to aflatoxin B1, diabetes and genetic disorders. Due to the high rate of recurrence and low detection rate at the curable stages, liver cancer has a poor prognosis. Statistics indicate that following hepatectomy, the survival rate of patients is 30–40% at five years (2). Therefore, a comprehensive knowledge of the pathogenesis of liver cancer is urgently required. The post-transcriptional expression of ≤30% of all protein-coding genes is estimated to be regulated by microRNA (miR/miRNA) (3). Furthermore, dysregulation of miRNA has been demonstrated to be a common characteristic of liver cancer (4). Therefore, studies that investigate the epigenetic modification of miRNAs and target genes may provide an improved understanding of the mechanism and action of liver cancer, and reveal novel approaches for curative cancer therapy.

## 2. miRNA

miRNAs are small, non-coding RNAs that vary in length from 19 to 25 nucleotides (nt). Previous studies have revealed that miRNAs are involved in various biological processes that underlie liver tumor formation. In particular, miRNAs negatively regulate gene expression at the post-transcriptional level. The biosynthesis of mature miRNAs involves three stages as follows: i) In the nucleus, primary transcripts are transcribed by RNA polymerase II, ii) cleaved by Drosha to produce shorter hairpin-shaped pre-miRNA and iii) following translocation to the cytoplasm by exportin5, the enzyme Dicer processes the pre-miRNA into a 19–25 nt miRNA duplex. One strand of this mature miRNA incorporates into the RNA-induced silencing complex (RISC) and binds to the 3′ untranslated region (3′UTR) of target mRNA. In this way, the degradation or translational repression of target mRNA is determined by complete complementary pairing with miRNA. Recent studies have demonstrated that miRNAs not only repress, but also activate, translation. Furthermore, several studies have revealed that miRNAs can activate translation by directly binding to the 3′UTR, the 5′UTR or the promoter region of the targeted mRNAs (5–7). The Argonaute 2 protein, an important component of the RISC, is believed to be involved in this process (8,9). However, the precise mechanisms involved in miRNA translational activation are unknown. Similar to protein-encoding genes, miRNAs are subjected to modulation at different levels, which includes regulation of miRNA biogenesis, metabolism and function. Certain studies have demonstrated that due to promoter region similarities, miRNAs are regulated in the same way as genes by transcription factors (TFs), enhancers, silencing elements and chromatin modifications (10). In summary, miRNAs are able to regulate gene expression by a variety of mechanisms. This dynamic regulation enables the cell to adapt to changes in the cellular environment, but may also explain the dysregulation of miRNA expression in certain types of cancer.

## 3. miRNA expression profiles in HCC

Since aberrant miRNA expression was first identified to be associated with the developmental lineage and differentiation state of tumors in 2005 (11), genome-wide miRNA expression profiling studies have been performed in cancer research. The study by Murakami *et al* (12) was the first to report that liver cancer exhibited an abnormal expression pattern of miRNAs. In total, four miRNAs (miR-92, miR-20, miR-18 and precursor miR-18), were revealed to be associated with the HCC differentiation states. Since then, a number of studies have confirmed that miRNAs possess important regulatory roles in hepatocarcinogenesis and malignant transformation. Furthermore, different subsets of miRNAs may be involved in the distinct biological characteristics of HCC. For example, epithelial cell adhesion molecule-positive human liver cancer stem cells are positively correlated with the expression of miR-181 (13). A study by Budhu *et al* (14)*,* which was based on the analysis of clinical sample data, identified a 20-miRNA metastasis signature that could predict primary HCC with venous metastases. In addition, a number of studies have indicated that certain miRNAs are associated with distinctive liver tumor risk factors. These include the observation that miR-26a-1 expression exhibits gender differences (15), that miR-96 is overexpressed in HBV-related HCC, and that the downregulation of miR-126 is associated with alcohol use (4). These studies provide an overview of the role of miRNAs in liver cancer biology, and may identify factors involved in the etiological diagnosis. Based on statistics from miRNA profiling, the present review screens and summarizes the functions of crucial miRNAs involved in the biological processes of HCC (4,12,16–34). Major aberrantly-expressed miRNAs and corresponding targets genes involved in HCC are listed in Table I.

## 4. Dysregulated miRNAs in HCC

The dysregulation of miRNA expression has been identified as a common characteristic of liver cancer (12). miRNAs are characterized as either proto-oncogenes or tumor suppressors, and are known to regulate the cell cycle, apoptosis and metastasis. In the present review, the roles of several miRNAs that are involved in the pathogenesis of HCC are reviewed.

### Oncogenic miRNA in HCC

Previous studies have revealed that miR-221/222, miR-21, miR-224 and miR-34a are consistently upregulated in HCC (4,23,33). These miRNAs promote tumor progression by impacting upon various cancer-related biological processes.

#### miR-221/222

miR-221 is considered as an ‘oncogenic’ miRNA that accounts for 71% of cirrhotic tissue HCC cases. The oncogenic effects of miR-221 are achieved via increased cellular proliferation and migration, and by the invasion of liver cancer *in vitro* and *in vivo* (33,35). Using a transgenic mouse model that overexpressed miR-221, Callegari *et al* (35) demonstrated that following treatment with diethylnitrosamine, ~50% of the mice developed spontaneous nodular liver lesions, and 100% experienced accelerated emergence of liver tumors. Further investigation has identified that during hepatocarcinogenesis, miR-221 promotes proliferation through direct targeting of CDKN1B/p27 and CDKN1C/p57 (36). miR-222 and miR-221 are homologous miRNAs that function similarly in the pathogenesis of HCC. A previous study identified that miR-221 and miR-222 induce tumor necrosis factor-related apoptosis-inducing ligand resistance and enhance cellular migration by modulating the expression of phosphatase and tensin homolog (PTEN) and TIMP3 (37). Additional molecules, including the pro-apoptotic protein B-cell lymphoma 2-modifying factor (BMF) (38) and the DNA damage-inducible transcript 4 (33), have been validated as targets of miR-22. In summary, miR-221 exerts its tumor-promoting function by regulating certain downstream genes that are involved in cancer-related processes.

#### miR-224

Compared with miR-221/222, miR-224 is a specific hallmark for HCC. The expression of miR-224 is undetectable in normal liver tissues, however, as liver disease progresses, the levels of miR-224 increase. A previous study revealed that miR-224 expression in liver cancer can be elevated >20-fold (4). Furthermore, miR-224 has been reported to promote the expression of p-PAK4 and MMP9 by targeting HOXD10, which contributes to HCC cell migration and invasion (39). In addition, miR-224 has been identified as part of the lipopolysaccharide, lymphotoxin α and tumor necrosis factor α (TNFα) inflammatory pathways, and as a link between cell migration and invasion in HCC. This is believed to be due to the multiple NFκB sites within the miR-224 promoter (40). A further study demonstrated that miR-224 deregulated the TNFβ signaling pathway in mouse granulosa cells by targeting SMAD4, which resulted in the dysregulation of proliferation and apoptosis (41). Therefore, miR-224 miRNA has multiple roles in the pathogenesis of liver cancer, and appears to be a useful biomarker for clinical diagnosis.

#### miR-21

Microarray analysis has identified miR-21 to be significantly elevated in HCC tumors and cancer cell lines. Through the silencing of PTEN, an important tumor suppressor, miR-21 promotes tumor cell proliferation, migration and invasion in HCC (34). Qiu *et al* (25) reported that miR-21 lowered programmed cell death 4 (PCD4) protein expression in HBV-related HCC, which weakened the tumor suppressive effects of PCD4 in liver cancer (25,26). In addition, mitogen-activated protein kinase-kinase 3 (MAP2K3) has been confirmed as one of the downstream targets of miR-21 that is involved in liver tumor cell proliferation (44).

### Tumor suppressive miRNAs in HCC

Certain subsets of miRNAs are reported to be silenced in human liver cancers, and therefore function as tumor suppressors. Screening from miRNA profiling most frequently identifies miR-122, miR-125a/b, miR-26, miR-199 and miR-375 as tumor suppressive miRNAs (12,19,22,27).

#### miR-122

miR-122 is a marker of hepatocyte-specific differentiation, and constitutes 70% of the total adult liver miRNA content (18,45). The study by Budhu *et al* (14) was the first to identify the association between miR-122 and HCC. Data obtained from HCC cases has indicated that >70% of patients exhibit low miR-122 expression (46). A further study demonstrated that a loss of miR-122 conferred metastatic migration and invasive properties to cells, increased the incidence of tumor recurrence and was predictive of poor patient outcomes (18). A number of genes involved in the regulation of tumorigenesis and cancer metastasis are targeted by miR-122. These include Pkm2 (47), HNF4A (18), RHOA, VEGF, HIF1A, vementin (18), a disintegrin and metalloprotease 10 (ADAM10), serum response factor, insulin-like growth factor 1 receptor and ADAM17 (48). Therefore, miR-122 has a central role in the suppression of HCC.

#### miR-375

An increasing number of studies have demonstrated the antitumor effects of miR-375. The study by Ladeiro *et al* (4) was the first to report that miR-375 is negatively associated with HCC progression, and that the silencing of miR-375 in HCC is characterized by a β-catenin-activating mutation (4). Our previous study also investigated the role of miR-375 in HCC and revealed that miR-375 expression levels were significantly downregulated in cancerous hepatocytes compared with normal primary human hepatocytes. The validation results of miR-375 expression in 60 pairs of HCC and adjacent non-tumor tissues were consistent with those in the cell lines. Further functional analysis revealed that an abnormal expression level of miR-375 decreased the rate of tumor cell proliferation and invasion, and induced G_1_ arrest and apoptosis. Furthermore, it was demonstrated that astrocyte elevated gene-1 (AEG-1), an important oncogene that is overexpressed in >90% of HCC cases, was a direct target of miR-375. Therefore, inhibition of AEG-1 restores miR-375 tumor suppressive function. In a mouse model, the administration of the miR-375 mimic, chol-miR-375, significantly suppressed the growth of hepatoma xenografts (49). In a further study, miR-375 was able to inhibit autophagy and impair the viability of HCC cells *in vivo,* and under hypoxic conditions in culture. Independent of PDK1/AKT/mTOR pathway modulation, miR-375 has demonstrated reduced hypoxia-induced autophagosome formation and autophagic flux in HCC cells by silencing the autophagy-associated gene, Atg7 (50). Therefore, the results of our previous study support the tumor suppressive role of miR-375 in HCC. In addition, Liu *et al* (32) identified that YAP1, another important oncogene, is the downstream target of miR-375 in liver tumors. Furthermore, miR-375 expression was revealed to be positively correlated with a decrease in HCC cell invasion and proliferation (35).

#### miR-125a/125b

By modulating tumor apoptosis and proliferation, miR-125a and miR-125b function as tumor suppressors. miR-125b promotes apoptosis by suppressing the anti-apoptotic molecules of the Bcl-2 family (5). Kim *et al* suggested that miR-125b suppresses SIRT7 and cyclin D1 expression, and induces p21-dependent G_1_ cell cycle arrest (28). In addition, miR-125b has been revealed to inhibit the expression of LIN28B2, an oncogene indicated to be involved in HCC cell growth (29).

#### miR-26a

A number of studies have demonstrated that miR-26a downregulation is significantly associated with HCC tumor recurrence, metastasis and a poor patient prognosis. As a tumor suppressor, miR-26a modulates a number of biological tumor processes. In a study by Kota *et al* (51), adeno-associated virus (AAV)-mediated delivery of miR-26a into a MYC-inducible liver cancer mouse model significantly suppressed tumor progression. In another study, Chen *et al* (26) revealed that the overexpression of miR-26a suppressed the G_1_/S transition and viability of tumor cells by inhibiting Era activity *in vitro* and *in vivo*. Furthermore, miR-26a has been demonstrated to suppress tumor growth and metastasis through the IL-6-Stat3 and HGF-cMet signaling pathways (15,52,53). In addition to the inhibition of gene expression, miR-26a has been reported to globally enhance miRNA biogenesis, in particular let-7, by directly downregulating Lin28B and Zcchc11 expression (25).

## 5. miRNA gene networks in HCC

miRNAs are important for the development and progression of liver cancer, however, the delivery of certain miRNAs fails to modulate the expression of putative target gene expression (18). The complicated regulatory networks between miRNA and mRNA are most likely to be involved in the process. In the present review, the crosstalk between miRNA and genes within putative oncogenic pathways and miRNA regulatory circuits is discussed, with the hope of understanding the roles of these components in the pathogenesis of HCC.

### miRNAs involved in inflammation-related pathways

HCC is an inflammatory form of cancer. In total, ~80% of patients (and particularly Chinese patients) develop HCC from chronic liver inflammation (54). A number of growth factors and cytokines that are secreted into the local tumor microenvironment promote the ability of tumor cells to invade and metastasize (55). The NF-κB and STAT3 signaling pathways are two inflammatory pathways that are involved in liver tumorigenesis and malignant transformation. A previous study demonstrated that enhanced IL-6-STAT3 signaling is positively correlated with the progression of liver tumors (15). Furthermore, Yang *et al* (52) revealed that miR-26a significantly decreased the expression of Bcl-2 and Mcl-1, two downstream effectors of STAT3 signaling. The same study identified that miR-26a directly suppressed IL-6 expression in cancer cells, and therefore inhibited IL-6-STAT3 signaling within liver tumors (52). Another miRNA, miR-124, has also been identified to modulate IL-6R expression. A STAT3 binding site is contained within the promoter regions of miR-24 and miR-629, which along with HNF4a, create an epigenetic circuit that bridges the gap between inflammation and tumorigenesis in HCC (56). Similarly, miR-155 activates STAT3 signaling, partly by the downregulation of the suppressor of cytokine signaling 1 protein (57). STAT3 has been revealed to induce the expression of certain miRNAs. Yang *et al* (58) identified that in cases of prostate cancer, STAT3 binds directly to the miR-21 promoter region in response to IFN, and suppresses IFN-induced apoptosis (48). Therefore, it can be concluded that miRNAs interact with multiple aspects of the STAT3-signaling pathway. From this perspective, a multi-target approach for miRNAs could be more beneficial for HCC treatment at the transcriptional and post-transcriptional levels (59).

NF-κB is an important transcription factor that links inflammation and tumorigenesis (60,61). A number of miRNAs are likely to be involved in the regulation of NF-κB signaling. In a study by Zhang *et al* (62), NF-κB bound to the putative promoter of miR-143 and conferred oncogenic functions in HBV-related HCC (51). In mice fed a choline-deficient L-amino acid-defined diet, miR-155 has also been identified to be regulated by NF-κB (62). Gene-network analyses have revealed that a low expression level of miR-26 is associated with the activation of the NF-κB and IL-6 signaling pathways (15). A high-throughput luciferase reporter screening identified a number of miRNAs that can directly target components of the NF-κB signaling pathway. Of these components, miR-195 suppressed cancer cell proliferation and migration by the downregulation of IKKa and TAB3, two NF-κB downstream effectors (64). Therefore, miRNAs are also able to regulate tumorigenesis by targeting multiple components involved in classic cancer-related signaling pathways. In this way, miRNAs can have a cascade effect on tumor progression.

### Significant miRNA regulatory circuits

An increasing number of studies have identified that miRNA-associated epigenetic circuits can induce tumor initiation and progression through auto-amplified, and permanent, oncogenic alterations (53,54). Classifying miRNAs according to regulatory networks enables an understanding of the gene expression alterations and biological functions that are involved in cancer. The entire epigenetic feedback circuit can occur with or without the absence of genetic abnormalities. Iliopoulos *et al* (64) demonstrated that activation of the oncoprotein Src triggered a positive inflammatory feedback loop in breast cancer cells, which involved NF-κB, Lin28, let7 and IL-6. This regulatory circuit explained the ability of immortalized breast cells to develop into a stable, transformed, cancer-like cell line within an inflammatory environment. Another study identified a targetable proinflammatory loop consisting of miR-24/miR-629/HNF-4α/miR-124/STAT3, and revealed that the transient silencing of HNF-4α in cell xenografts maintained low levels of HNF-4α in liver cancers (56). Furthermore, the study demonstrated that miR-124 delivery was sufficient to limit tumor growth. Therefore, it appears to be possible to block essential feedback loops in hepatocarcinogenesis with specific miRNA delivery. Recently, Wang *et al* identified a feedback loop that silenced the tumor suppressive miRNA, miR-101, during hepatocarcinogenesis. Furthermore, the study demonstrated that c-myc recruited the EZH2-containing PRC2 complex, and epigenetically repressed miR-101 expression. This repression of miR-101 expression in turn inhibited EZH2 and EED, two subunits of the PRC2 complex (54). In conclusion, the interaction between miRNAs and gene expression is a complicated regulatory network that requires further investigation.

## 6. Clinical applications of miRNAs in HCC

As extensively reviewed, the abnormal expression of miRNAs has been demonstrated in a variety of cancers. Over the course of cancer progression, miRNAs participate in a number of biological processes, including proliferation, apoptosis, invasion and metastasis. Therefore, studies concerning miRNAs appear to show a novel perspective for cancer diagnosis and treatment.

miRNA expression profiles provide valuable information that allows for the discrimination between different liver cancer malignancies. By comparing miRNA expression profiles in 60 pairs of HCC and corresponding non-cancerous tissues, Wei *et al* (66) demonstrated a 30-miRNA signature that could distinguish liver cancer from non-tumorous liver tissues in the training set, with a 99.2% accuracy. In total, 20 miRNAs were revealed to be associated with the survival rates of the HCC patients, and were regarded as independent prognostic predictors (66). A second HCC miRNA profiling study by Budhu *et al* (14), which included a 482-human miRNA microarray, revealed that a metastasis-related 20-miRNA signature could significantly discriminate liver cancer from venous metastatic and non-metastatic cancer tissues, with an accuracy of 76%. The 20-miRNA signature could therefore be used to calculate the risk of metastasis and recurrence in HCC patients, and act as a cancer-related biomarker of metastasis (14). However, during recent years, the use of circulating miRNAs as a potential tool for HCC detection has become an emerging area of study. Circulating miRNAs that are released from cancerous tissues are stable and readily available for clinical analysis, and therefore may be useful for the first-line detection of cancer. Recently, a number of studies have identified that altered expression profiles of serum miRNAs in HCC patients have contributed to the improvement of sensitivity and specificity of liver cancer diagnosis (67,68). Tomimaru *et al* (67) reported that the level of miR-21 was increased in patients with HCC compared with patients with chronic hepatitis and healthy controls. Furthermore, the level of miR-21 in cases of HCC was higher compared with that of α-fetoprotein, a traditional diagnostic marker of HCC. Other serum miRNAs, such as miR-222 and miR-223, have also been identified to be upregulated in HCC patients carrying HBV or HCV (68). The levels of certain miRNAs, such as miR-1, do not differ significantly between cases of HCC and liver cirrhosis, but are independently associated with overall survival. The serum levels of miR-122, however, alter according to different clinical chemistry parameters in liver cancer (69). Therefore, miRNAs may provide a useful tool to stratify the prognosis and monitor the follow-up of patients with HCC.

To date, a number of studies have identified miRNAs as therapeutic targets for HCC, which may provide a novel approach for the treatment of cancer. In patients with liver cancer, approaches based on the modulation of miRNA activity focus on inhibiting oncogenic miRNAs and restoring the activity of tumor-suppressor miRNAs. A study by Park *et al* (70) revealed that the intravenous administration of a cholesterol-modified isoform of anti-miR-221 oligonucleotide, into an orthotopic mouse model of liver cancer, reduced tumor cell proliferation and increased markers of apoptosis. This suggested that the targeted inhibition of miRNA contributed to the successful treatment of HCC. Another method that has potential value for the treatment of HCC is miRNA replacement therapy. Using mouse MYC-induced liver tumors, Kota *et al* (51) identified that compared with other miRNAs, miR-26a demonstrated the most notable change in expression. Furthermore, the restoration of miR-26a expression using an AAV delivery system in the same model inhibited proliferation and promoted cancer cell apoptosis, but did not induce apoptosis in the non-malignant hepatocytes (51). An *in vivo* study by Ma *et al* (71) revealed that the upregulated expression of miR-122 in metastatic Mahlavu and SK-HEP cells inhibited intrahepatic metastasis, and led to a decreased rate of tumorigenesis and angiogenesis. Therefore, the development of miRNA-based therapies could be of potential value for future HCC treatment regimens.

## 7. Conclusions

miRNAs exert tumor-promoting or antitumor activity by regulating the translation rate of >60% of the protein-coding genes (72). A number of studies have revealed that miRNAs have a pivotal role in a variety of biological processes that are involved in the pathogenesis of liver cancer. In recent decades, studies have attempted to understand the role of miRNAs in liver tumors. These studies have demonstrated that certain miRNAs are associated with feedback loops, and that others are involved in classic cancer-related pathways. Furthermore, the associations between miRNA alterations and the cancer microenvironment has been investigated, all of which are important for liver tumorigenesis and cancer metastasis. In addition, certain studies have examined the value of miRNA-based diagnosis and therapy for HCC. Despite progress in this area of research, certain issues are yet to be addressed. Firstly, it is necessary to ensure that miRNA delivery systems are guaranteed to be effective and non-toxic to humans. Secondly, since miRNAs have a significant role in the pathogenesis of HCC, the generation of regulatory networks that consist of genes and miRNAs in liver tumors should be considered; in this way the biological behaviors of cancer could be comprehensively recognized.

## Figures and Tables

**Table I:** Major aberrantly-expressed microRNAs and target genes in heptocellular carcinoma.

miRNA	Reference	Targets
Downregulated		
miR-122	(4), (16), (17), (18), (19), (20), (21)	Pkm2, HNF3B, RHOA, ADAM10, ADAM17, Cyclin G1, IGF1R, Bcl-w
miR-200a	(12), (17), (19), (22), (23)	Zeb1, Zeb2, E-cadherin
miR-199a	(12), (22), (23), (24)	Kras, TIMP3, Fibronectin
miR-26	(15), (25), (26), (27)	Lin28B, Zcchc11, Era, cyclin D2, cyclin E2, MTDH, EZH2, NF-κB pathway
miR-125a	(12), (19), (22), (23)	SIRT7
miR-125b	(19), (28), (29)	LIN28B, mcl-1, IL6R, Bcl2
miR-375	(27), (30), (31), (32)	AEG1, YAP, ATG7
Upregulated		
miR-221	(19), (20), (23), (33)	p27, p57, Bmf, PTEN, TIMP3, DDIT4
miR-34a	(14), (16), (23), (33)	Bcl2, CD44, SIRT1
miR-222	(4), (19), (20), (23), (24), (33)	p27, PTEN, TIMP3, PPP2R2A
miR-21	(4), (16), (23), (33), (34)	MAP2K3, PTEN, RhoB
miR-224	(4), (12), (14), (17), (19), (22), (23), (24)	Smad4, HOXD10, API-5, NF-κB pathway

miR/miRNA, microRNA.

## Abbreviations

miRNA: microRNA
RISC: RNA-induced silencing complex
3′UTR: 3′ untranslated region
HCC: hepatocellular carcinoma
BMF: pro-apoptotic protein B-cell lymphoma 2-modifying factor
PTEN: phosphatase and tensin homolog
ADAM: a disintegrin and metalloprotease
AEG1: astrocyte elevated gene-1
Atg7: autophagy-related protein 7
